# Influence of Si wall thickness of CsI(Tl) micro-square-frustums on the performance of the structured CsI(Tl) scintillation screen in X-ray imaging

**DOI:** 10.1038/s41598-022-12673-9

**Published:** 2022-05-24

**Authors:** Zhixiang Sun, Mu Gu, Xiaolin Liu, Bo Liu, Juannan Zhang, Shiming Huang, Chen Ni

**Affiliations:** Shanghai Key Laboratory of Special Artificial Microstructure Materials and Technology, School of Physics Science and Engineering, Tongji University, Shanghai, 200092 China

**Keywords:** Imaging techniques, Optical materials and structures

## Abstract

To improve the detection efficiency of the structured scintillation screen with CsI(Tl) micro-square-frustums based on oxidized Si micropore array template in the case of a period as small as microns, the influence of Si wall thickness of the CsI(Tl) micro-square-frustums on the performance of the structured screen in X-ray imaging was investigated. The results show that when CsI(Tl) at the bottom of the screen is structured, the detective quantum efficiency (DQE) improves at almost all spatial frequency as the top thickness of the Si wall t_Si_ decreases. However, when CsI (Tl) at the bottom of the screen is not structured, the DQE becomes better at low-frequency and worse at high-frequency as t_Si_ decreases. The results can provide guidance for optimizing t_Si_ according to the comprehensive requirements of detection efficiency and spatial resolution in X-ray imaging.

## Introduction

CsI(Tl) scintillation screen has been widely used in X-ray imaging because of its high light yield and detection efficiency^1,2^. To enhance detection efficiency, the thickness of the scintillation screen must be increased. As a result, the spatial resolution of X-ray imaging is reduced due to the lateral spread of scintillation light. To overcome this problem, the scintillation screen with columnar structure was developed. The scintillation light can be guided to propagate along the column channels and the lateral spread is restrained. The CsI(Tl) scintillation screens with needle-like columnar have been developed by vapor deposition method and the spatial resolution of X-ray imaging has been improved^3–5^. However, the improvement is imperfect and the lateral spread of the scintillation light between adjacent columns is still considerable because the columns are close to each other. To further suppress the lateral spread of scintillation light, the structured CsI(Tl) scintillation screen based on oxidized silicon micropore array template has been developed by filling the scintillator into the template^6–11^. The Si walls between the adjacent microcolumns are used to absorb the incoming scintillation light to prevent its lateral spread, and the SiO_2_ reflective layers grown on each side of the Si walls are used to guide the scintillation light with an incident angle *θ* larger than the critical angle *θ*_c_ to propagate along the column channels based on the principle of total reflection of light. The thickness of the SiO_2_ layers should not be less than 0.3 μm in consideration of the optical tunneling effect^12^. It has been reported that the spatial resolution of X-ray imaging has reached over 100 lp/mm by using the structured CsI(Tl) and CsI scintillation screens with microcolumn array period of 4 μm, respectively^10,11^. To achieve an effective optical isolation, the pore wall of the template needs to have a certain thickness, and is generally considered to be not less than 1 μm^13^. Therefore, when the array period of the structured scintillation screen is as small as microns, the detection efficiency of the screen will be restricted because the ratio of the filled CsI(Tl) area to that of the entire structured scintillation screen will be limited. To improve the detection efficiency, the structured CsI(Tl) scintillation screen with micro-conical-frustums was proposed^14^ and the frustums pore can be prepared by metal-assisted chemical etching^15,16^. The new structure with a cone angle of 2.4° and array period of 4 μm can increase the bottom light output by 14.5%. Compared with the columns, the frustums can guide more scintillation light into the total reflection and make it propagate along the channel.

This work intends to study the influence of the Si wall thickness on the X-ray imaging performance of the structured scintillation screen based on CsI(Tl) micro-square-frustum array to explore the possibility of further improving the detection efficiency of the scintillation screen. The performance of the scintillation screen was mainly characterized by the bottom light output (BLO), modulation transfer function (MTF) and detective quantum efficiency (DQE). The simulation was carried out by the Geant4 Monte Carlo simulation toolkit^17,18^, finite-difference time-domain method^19^ and Monte Carlo ray-tracing method^20^. The results can provide guidance for optimizing Si wall thickness and improving the performance of the structured CsI(Tl) screen in X-ray imaging.

## Structure and methods

### Screens structure

**Figure 1:**
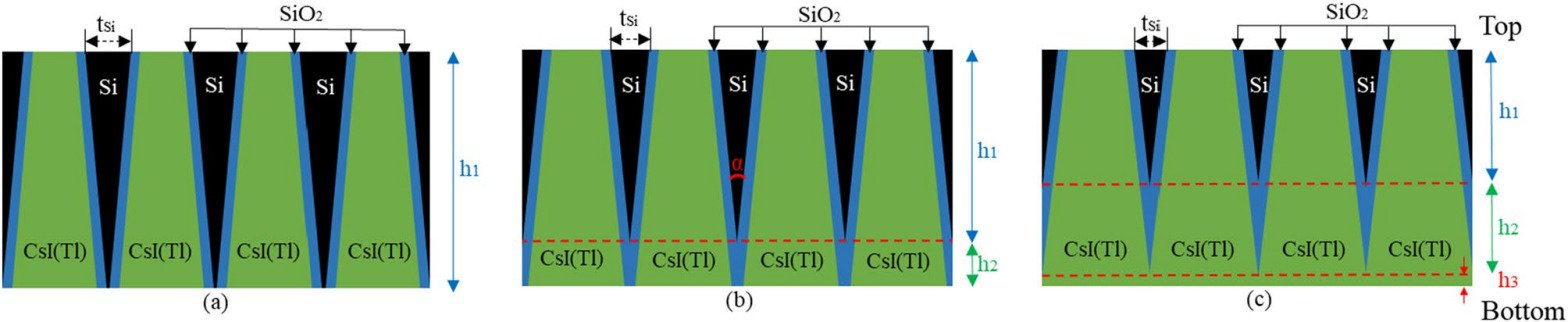
Side view of the CsI(Tl) micro-square-frustums structured scintillation screen with different t_Si_. As t_Si_ decreases, the screen from top to bottom presents (**a**) one structural layer; (**b**) two structural layers; (**c**) three structural layers.

**Figure 2:**
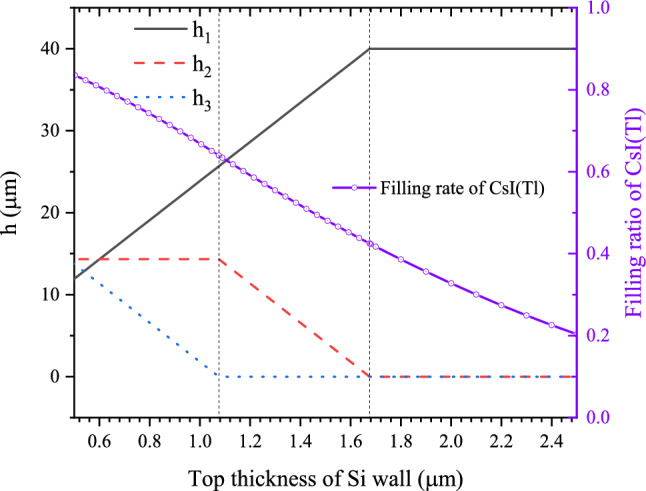
Variation of h_1_, h_2_, and h_3_ with t_Si_, and variation of the filling ratio of CsI(Tl) with t_Si_.

The structured scintillation screens are composed of oxidized silicon mircopore array templates filled with CsI(Tl) scintillator. The mircopores are 40 μm high, in a shape of a square-frustum, and arranged in a square array of 4.0 μm period, as shown in Fig. 1. The cone angle α of the micro-square-frustums was set to 2.4°, which is the maximum cone angle in the literature^14^. The walls between the micro-square-frustums consist of Si walls covered with a 0.3 μm thick SiO_2_ layer on each side. From the side view of the structured scintillation screen, there can be three structural layers from top to bottom. They are: (a) The micro-square-frustums are divided by Si walls covered by SiO_2_ layer on each side, and the height of the structural layer is represented by h_1_; (b) The micro-square-frustums are divided only by the SiO_2_ layers, and the height of the structural layer is represented by h_2_; (c) There is no separation wall between the micro-square-frustums, and the height of the structural layer is represented by h_3_. Figure 2 shows the variation of h_1_, h_2_, and h_3_ with t_Si_ (top thickness of Si wall). When t_Si_ > 1.68 μm, there is only the first structural layer and the thickness difference between the top and bottom of the Si wall is 1.68 μm. When 1.08 μm < t_Si_ < 1.68 μm, there are the first and second structural layers. When t_Si_ < 1.08 μm, all three structural layers exist. The variation of the filling ratio of CsI(Tl) with t_Si_ is also shown in Fig. 2. It can be seen that the ratio rises quickly as t_Si_ decreases.

### BLO, MTF, and DQE

The bottom light output (BLO) is defined as the average number of scintillation photons generated by each X-ray photon that can reach the bottom of the structured scintillation screen. The BLO depends on the scintillation photon yield generated by CsI(Tl) and the light guide efficiency of the scintillation screen.

The modulation transfer function (MTF) can be used to characterize the resolution capability of an X-ray imaging system. The standard edge measurement method^21^ was used to calculate the MTF. An edge spread function (ESF) was derived from an edge phantom, which was the response of the X-ray imaging system to an opaque object with a straight edge. The MTF was calculated by Fourier transform of a line spread function (LSF), which was deduced from the differential of the ESF along the direction perpendicular to the straight edge.

1 $${\text{LSF}}\left( x \right) = \frac{{\text{d}}}{{{\text{d}}x}}{\text{ESF}}\left( x \right)$$

2 $$\text{MTF}\left( {\text{f}} \right) = \left| {\frac{1}{{\sqrt {2\pi } }}\int_{ - \infty }^{\infty } {\text{LSF}\left( {\text{x}} \right){\text{e}}^{{ - 2\pi {\text{ifx}}}} \text{d}{\text{x}}} } \right|$$

where *x* is the position coordinate perpendicular to the straight edge direction, *f* is the spatial frequency in *x* direction. The MTF was normalized at zero frequency. The spatial resolution of the detector is defined by the spatial frequency where the MTF = 0.1.

The detective quantum efficiency (DQE) is the frequency-domain spectral representation of the signal-to-noise characteristics of a given detector configuration. According to the International standard IEC 62220-1^22^ it can be expressed as

3 $${\text{DQE}}\left( f \right) = \frac{{{\text{Q}} \cdot {\text{MTF}}^{2} \left( f \right)}}{{{\text{NPS}}\left( f \right)}}$$

where Q represents the X-ray photon flux incident on the scintillation screen and NPS is the output noise power spectrum of the X-ray imaging detector calculated using a uniformly exposed image. The exposed image was divided into several 256 × 256 pixel regions of interest (ROIs) that overlapped half the size of one another. The 2D NPS is given by

4 $${\text{NPS}}(u,v) = \frac{\Delta x \cdot \Delta y}{{M \cdot 256 \cdot 256}}\sum\limits_{m = 1}^{M} {\left| {\sum\limits_{i = 1}^{256} {\sum\limits_{j = 1}^{256} {({\text{I}}(x_{i} ,y_{j} ) - {\text{S}}(x_{i} ,y_{j} )) \cdot e^{{ - 2\pi i(ux_{i} + vy_{j} )}} } } } \right|^{2} }$$

where *x* and *y* are the position coordinate of the pixel, *i* and *j* are the sequence number of the pixel, *u* and *v* are spatial frequencies in *x* and *y* directions, respectively, *M* is the number of ROIs, Δ*x* and Δ*y* are the pixel sizes, I(*x*, *y*) is the pixel value, and S(*x*, *y*) is the optionally fitted two-dimensional polynomial. The 1D NPS was obtained as the average of the NPS(*u*, *v*) data in the 7 rows or columns on either side of the corresponding axis over the frequency interval (*f* − 0.5 × *f*_int_ ≤ *f* ≤ *f* + 0.5 × *f*_int_), where *f* = (*u*^2^ + *v*^2^)^1/2^ and *f*_int_ = 0.01/pixel pitch (mm).

### Simulation methods

The performance of the structured CsI(Tl) scintillation screen based on oxidized Si template in X-ray imaging was simulated using the version 10.0 of Geant4 Monte Carlo simulation toolkit^17,18^, finite-difference time-domain method^19^ and Monte Carlo ray-tracing method^20^. The interaction of X-ray with scintillation screen, the light generation process, transmission process and detection process need to be considered in the simulation. The Geant4 Monte Carlo simulation toolkit including the photoelectric effect, Compton scattering, coherent scattering, ionization, multiple scattering, bremsstrahlung and de-excitation was used to simulate the interaction of X-ray with scintillation screen, and the light generation process. The incidence of each X-ray photon in the simulation was treated as an event. The number of events was set to stabilize the results such as BLO and MTF. The event ended when all primary and secondary particles had disappeared. The transmission process of the scintillation light such as optical absorption and boundary processes was performed using the Monte Carlo ray-tracing method and finite-difference time-domain method because the ray optics cannot simulate the surface process of the scintillation light when the thicknesses of SiO_2_ and Si walls are less than or close to the wavelength of the light. For the Monte Carlo ray-tracing simulation, the probabilistic split rays trace mode was used. It means that the reflected and transmitted rays are traced. The transmittance and reflectance were computed based on Fresnel equations. The attenuation of ray power was computed based on the absorption lengths of the materials. The relative ray power threshold was set to 1%, which means that the ray will be terminated if the ray power is less than 1% of the original power. The ray would also be terminated if it was outside the scintillation screen. For the finite-difference time-domain simulation, the scintillation light incident on the surface of SiO_2_ layer from CsI(Tl) was represented by plane waves. The transverse electric wave and transverse magnetic wave were considered. The unit cell sizes in the directions parallel and perpendicular to the SiO_2_ interface were set to 1 and 3 μm, respectively. The mesh size was set to 1/34 of the wavelength. The boundary conditions were set as periodic boundary conditions in the direction perpendicular to the SiO_2_ interface, and perfectly matched boundary conditions in the direction parallel to the SiO_2_ interface.

The following parameters were needed in the simulation. The light yield and X-ray excited luminescence spectrum of CsI(Tl) were cited from Refs.^1,23^. The optical attenuation coefficient and refractive index of CsI(Tl) were cited from Ref.^24^. The refractive indices, optical attenuation coefficients of Si and SiO_2_ were cited from Refs.^25–27^. The energy of the X-ray beam incident uniformly and vertically from above the scintillation screen is 20 keV. The intensity of X-rays incident on the screen was set to 4.0 × 10^6^ mm^−2^. An ideal photodetector was placed at the bottom of the structured scintillation screen and a thin layer of silicone oil with a refractive index 1.465 is coupled between the scintillation screen and detector.

The same method has been used to study the performance of a CsI(Tl) scintillation screen with a dual-periodic structure based on oxidized silicon micro-pore array template in X-ray imaging^28^. The validity of the simulation method has been confirmed in our related preliminary experiments, which will be presented in our literature in the near future.

## Results and discussion

### Bottom light output

**Figure 3:**
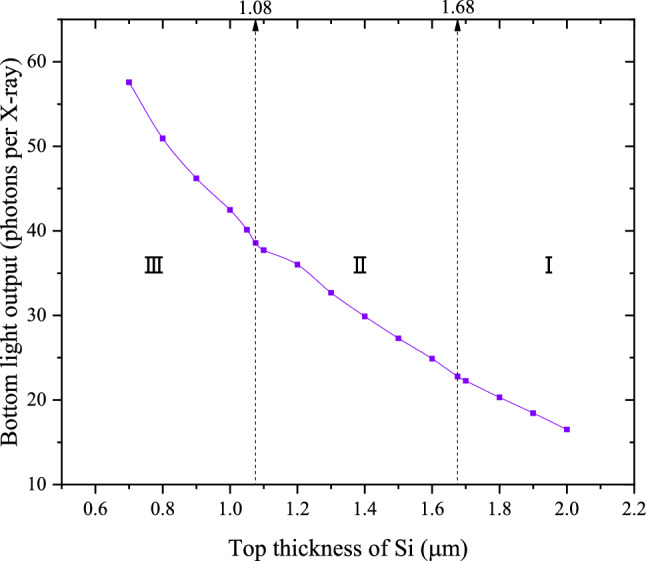
Bottom Light output of the structured CsI(Tl) screen varies with the top thickness of Si wall.

The bottom light output of the structured CsI(Tl) scintillation screen varies with the top thickness of the Si wall, as shown in Fig. 3. When t_Si_ decreases from 2.00 to 0.70 μm, the BLO increases by 3.47 times. Specifically, when t_Si_ decreases from 2.00 to 1.68 μm, the BLO increases by 38%; when t_Si_ decreases from 1.68 to 1.08 μm, the BLO increases by 69%; when t_Si_ decreases from 1.08 to 0.70 μm, the BLO increases by 49%. That is, when the second structural layer appears, the increase rate of BLO with the decrease of t_Si_ is almost the same as that when only the first structural layer appears; when the third structural layer appears, the increase rate of BLO with the decrease of t_Si_ is higher than that when the first structural layer appears or when the first and second structural layers appear simultaneously.

The main reason for the increase in BLO is the corresponding increase in the ratio of the filled CsI(Tl) area to that of the entire structured scintillation screen. The reduced optical isolation performance caused by the thinning of the Si wall is the secondary reason for the enhancement of BLO, which accounts for about 15.4%-20.7% of the increment.

**Figure 4:**
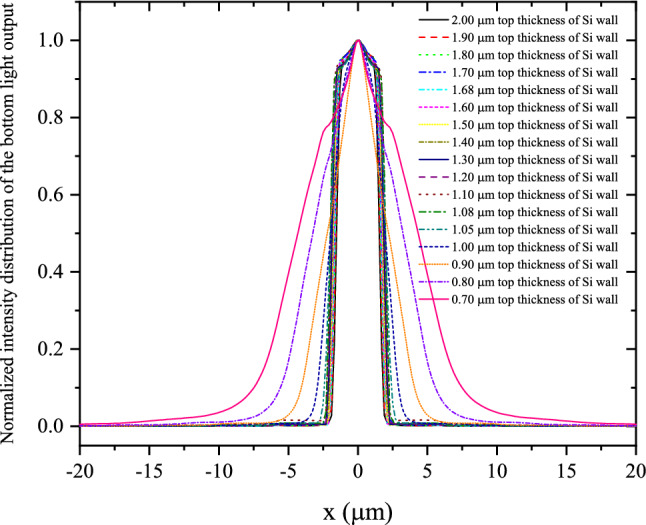
Normalized intensity distributions of BLOs of the structured CsI(Tl) screens with different t_Si_.

**Figure 5:**
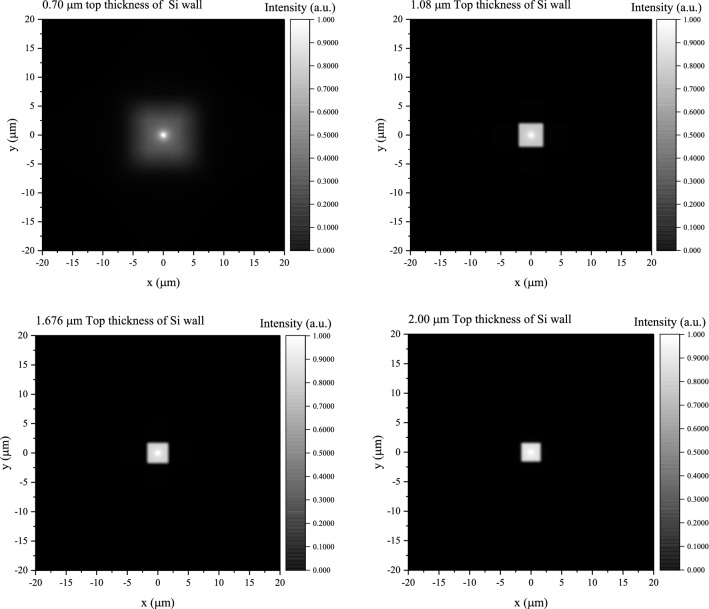
Normalized two-dimensional intensity distribution of BLO with the t_Si_ of 0.70, 1.08, 1.68 and 2.00 μm, respectively.

The normalized intensity distributions of BLOs of structured CsI(Tl) screens with different t_Si_ were simulated when an narrow X-ray beam is incident along and on the central axis of a scintillation micro-square-frustum. Figure 4 gives the distributions on the horizontal axis with the centre of the micro-square-frustum as the origin. It can be seen that when t_Si_ decreases from 2.00 to 1.08 μm, the distribution is relatively concentrated without obvious change. This shows that the optical isolation performance when both the first and second structural layers, as mentioned in Fig. 1, exist in the scintillation screen is basically the same as the performance when only the first structural layers exists. However, when t_Si_ is less than 1.08 μm, which is when the third structural layer also appears, the distribution becomes more and more diffuse as t_Si_ decrease. That is, the optical isolation performance becomes worse as t_Si_ decreases. The normalized two-dimensional intensity distributions of BLOs with t_Si_ of 0.700, 1.08, 1.68 and 2.00 μm are given in Fig. 5, so as to more intuitively show the influence of the top thickness of Si wall on the optical isolation.

### Modulation transfer function

**Figure 6:**
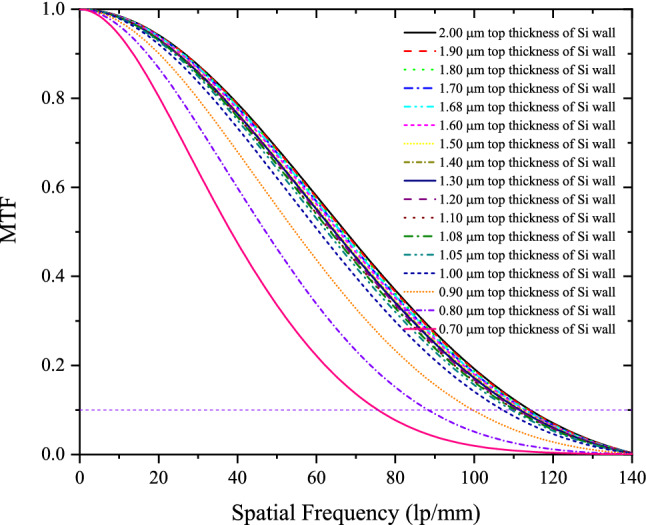
MTFs for the structured CsI(Tl) screens with different t_Si_.

**Figure 7:**
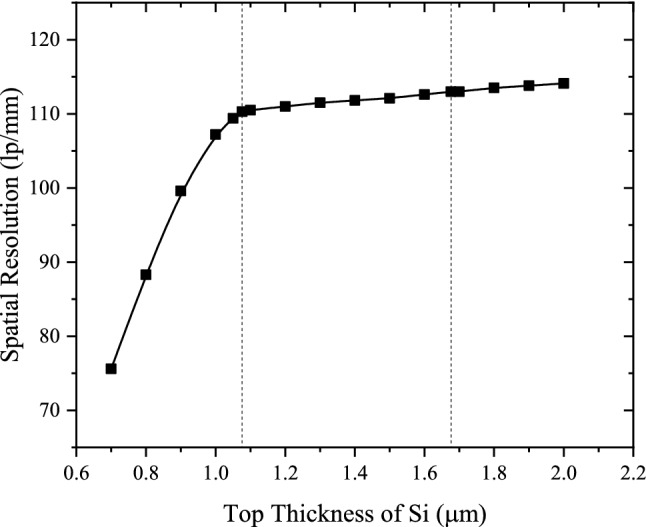
Spatial resolutions for the structured CsI(Tl) screens with different t_Si_.

The simulated MTFs for the structured CsI(Tl) with different t_Si_ and the derived spatial resolutions of X-ray imaging are shown in Figs. 6 and 7, respectively. It can be seen that when t_Si_ decreases from 2.00 to 1.08 μm, the MTF changes very little, and the spatial resolution remains good, only decreasing from 114 lp/mm to 110 lp/mm. However, when t_Si_ is less than 1.08 μm, the MTF and spatial resolution deteriorate rapidly with the decrease of t_Si_. This is because the third structural layer without optical isolation appears and its height gradually increases accordingly. The variations of MTF and spatial resolution with t_Si_ are completely in accordance with the variation of the normalized intensity distribution of BLO. When t_Si_ decreases from 1.08 to 0.70 µm, h_3_ increases from 0.00 to 8.97 µm, and the corresponding spatial resolutions decreases from 110 to 76 lp/mm.

### Detective quantum efficiency

**Figure 8:**
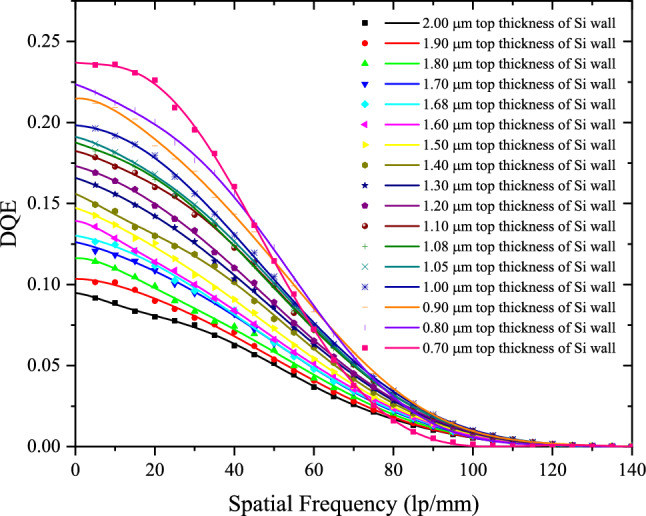
DQEs for the structured CsI(Tl) screens with different t_Si_.

**Figure 9:**
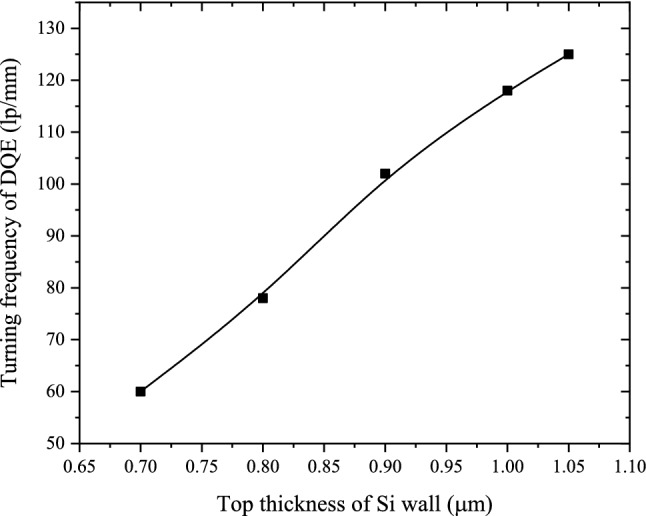
Variation of the turning frequency of DQE from rising to falling with t_Si_.

The simulated DQEs for the structured CsI(Tl) screens with different t_Si_ are shown in Fig. 8. When t_Si_ decreases from 2.00 to 1.08 μm, the DQE increases at almost all frequency. This is because the BLO increases and MTF does not change significantly. However, when t_Si_ is less than 1.08 μm, the DQE increases at low-frequency and decreases at high-frequency with the decrease of t_Si_. The reason is that the DQE in the low-frequency region is mainly affected by the X-ray detection efficiency and light guide efficiency, but as the frequency increases, the influence of the MTF on the DQE becomes important. Compared with the DQE when t_Si_ is 1.08 μm, the variation of the turning frequency of DQE from rising to falling with t_Si_ is shown in Fig. 9. It decreases as t_Si_ decreases. The simulated results can give the optimal t_Si_ according to the comprehensive requirements of detection efficiency and spatial resolution in X-ray imaging.

## Conclusions

In order to improve the bottom light output and spatial resolution of the structured scintillation screen composed of CsI(Tl) micro-square-frustum array, the influence of the Si wall thickness of the micro-square-frustums on the performance of the structured screen in X-ray imaging was studied. The array is a square array with a period of 4 μm. The CsI(Tl) micro-square-frustums are 40 μm high, the cone angle of the frustums is 2.4°, and the top thickness of the Si wall between the two nearest-neighbor frustums can vary from 0.70 to 2.00 µm. If viewed from the side of the structured scintillation screen, there can be three structural layers from top to bottom. They are: (1) The micro-square-frustums are divided by Si walls covered by SiO_2_ layer on each side; (2) The micro-square-frustums are divided only by the SiO_2_ layers; (3) There is no separation wall between the micro-square-frustums. When t_Si_ > 1.68 μm, there is only the first structural layer. When 1.08 μm < t_Si_ < 1.68 μm, there are the first and second structural layers. When t_Si_ < 1.08 μm, all three structural layers exist. The performance of the structured CsI(Tl) screen in X-ray imaging was characterized by the bottom light output (BLO), modulation transfer function (MTF) and detective quantum efficiency (DQE). The simulation was carried out by the Geant4 Monte Carlo simulation toolkit, finite-difference time-domain method and Monte Carlo ray-tracing method.

When t_Si_ decreases from 2.00 to 1.08 μm, the BLO of the structured CsI(Tl) screens increases by 233%. The normalized intensity distribution of the BLO is relatively concentrated without obvious change. The results show that the BLO increases with the decrease of t_Si_, and the optical isolation performance when both the first and second structural layers exist in the scintillation screen is basically the same as the performance when only the first structural layers exists. Meanwhile, the MTF changes very little, and the spatial resolution remains good which is only decreasing from 114 lp/mm to 110 lp/mm. Based on the performance of the BLO and MTF, the DQE increases as t_Si_ decrease at almost all frequency. However, when t_Si_ decreases from 1.08 to 0.70 μm, the third structural layer also appears and thickens. In this case, the BLO of the structured CsI(Tl) screens increases by 49%, but the distribution becomes more and more diffuse. Meanwhile, the MTF deteriorates rapidly, and the spatial resolution decreases from 110 lp/mm to 76 lp/mm. As a result, the DQE increases at low-frequency and decreases at high-frequency with the decrease of t_Si_. The reason is that the DQE in the low-frequency region is mainly affected by the X-ray detection efficiency and light guide efficiency, but as the frequency increases, the influence of the MTF on the DQE becomes important. Compared with the DQE when t_Si_ is 1.08 μm, the turning frequency of DQE from rising to falling decreases as t_Si_ decreases.

In conclusion, if there is only the first structural layer or the first and second structural layers in the structured scintillation screen composed of CsI(Tl) micro-square-frustum array, the smaller t_Si_, the better X-ray imaging performance of the screen. However, If there is also the third structural layer in the structured CsI(Tl) scintillation screen, the BLO increases and the MTF deteriorates as t_Si_ decreases. As a result, the DQE increases at low-frequency and decreases at high-frequency with the decrease of t_Si_. In this case, if a higher image signal-to-noise ratio on a large scale is required, a smaller t_Si_ is preferred, and if a higher image spatial resolution on a small scale is required, a larger t_Si_ is preferred. The results can provide guidance for optimizing t_Si_ according to the comprehensive requirements of detection efficiency and spatial resolution in X-ray imaging.

## Data Availability

The data generated or analysed during this study are included in this published article or its supplementary file.
